# Spatio-Temporal Influence on the Distribution of Forensically Relevant Blowflies (Diptera: Calliphoridae) in Gyeongsangnam-do, South Korea

**DOI:** 10.3390/insects15070536

**Published:** 2024-07-17

**Authors:** Hyeon-Seok Oh, In-Seong Baek, Min-Gyu Kang, Sang-Hyun Park

**Affiliations:** Department of Biomedical Sciences, Kosin University, Wachi-ro 194, Yeongdo-gu, Busan 49104, Republic of Korea; tjr3863@naver.com (H.-S.O.); is6630@naver.com (I.-S.B.); alsrb0886@gmail.com (M.-G.K.)

**Keywords:** forensic entomology, Calliphoridae, season, habitat, bait trap, community composition

## Abstract

**Simple Summary:**

Research into the community composition and succession of blowflies can provide valuable insights into the timing and environmental conditions of colonization events, enhancing the accuracy of forensic analyses. Blowfly species diversity and distribution are influenced by various environmental factors such as season, habitat, and geographical location, which in turn affect their behavior and colonization patterns. The researchers surveyed blowfly populations across four distinct regions over the course of a year, examining how factors like season, habitat, and location influenced the blowfly community. The findings showed significant seasonal variations, emphasizing the impact of environmental conditions on blowfly behavior and colonization patterns. This research provides important regional data on forensically relevant blowfly species, which can help improve the accuracy of estimating the time and circumstances of death during criminal investigations. By enhancing our understanding of blowfly ecology, this work also contributes to broader scientific knowledge about decomposition processes and the role of insects in ecosystem functioning, ultimately benefiting crime scene investigations and advancing the field of forensic science.

**Abstract:**

The study of blowfly (Diptera: Calliphoridae) biodiversity and distribution is crucial for forensic investigations. Abiotic and biotic factors, such as season and habitat type, have a significant impact on blowfly populations. However, only a few forensic entomology studies have been conducted in South Korea, particularly in the Gyeongsangnam-do region. To address this, an extensive year-long survey was conducted to analyze the compositions, habitat preferences, distribution, and seasonal abundance of forensically relevant blowflies in urban and forested habitats of Gyeongsangnam-do, with sampling conducted twice a month using mouse carcass-baited traps set for 48 h each time. A total of 3470 adult blowflies were recorded, encompassing five genera and 13 species, with a noted absence of specimens during the winter months. The predominant species was *Lucilia porphyrina*, accounting for 37.2% of the total sample, followed by *Chrysomya pinguis* (27.6%), *Lucilia sericata* (7.6%), and *Lucilia illustris* (7.1%). The species composition was consistent across all surveyed regions; however, seasonal variation in species diversity was evident, with a peak in spring and a decline in summer. Notably, certain species exhibited clear preferences for either urban (*Calliphora calliphoroides* and *L. sericata*) or forested habitats (*L. porphyrina* and *Ch. pinguis*). This pioneering study elucidates the diverse blowfly communities in Gyeongsangnam-do, highlighting significant seasonal and habitat-dependent variations. These findings enrich our understanding of blowfly ecology in this region, offering valuable insights for forensic applications and underscoring the necessity for ongoing entomological surveillance and research.

## 1. Introduction

In medico-legal entomology, the duration of insect colonization on human or animal remains at a crime scene provides critical insights for accurately estimating the postmortem interval (PMI). When conditions facilitate insect access to a carcass, insects typically begin colonization during the initial stages of decomposition. This process allows for the precise estimation of the Time of Colonization (TOC) or the minimum Postmortem Interval (mPMI) [1,2]. Medico-legal entomologists estimate the mPMI by analyzing larval growth duration as an indicator of the time since death [3,4,5] or by examining the succession patterns of insects during different stages of decomposition [6,7]. Additionally, they strive to understand the decomposition process through changes in the weight and physical characteristics of the remains [8,9].

The blowfly (Diptera: Calliphoridae) plays a significant role as a primary decomposer of carcasses post-mortem, contributing to the ecological processes of decay [10,11,12,13]. Research into the community composition and succession of blowflies can provide valuable insights into the timing and environmental conditions of colonization events, enhancing the accuracy of forensic analyses [14,15]. Blowfly species diversity and distribution are influenced by various environmental factors such as temperature, habitat, and geographical location, which in turn affect their behavior and colonization patterns [16,17,18,19]. The behavior of blowflies, including their approach patterns to carcasses, is another critical aspect of medico-legal entomology. Seasonal variations and environmental conditions significantly influence these behaviors, affecting the timing and nature of colonization [20,21,22].

Localized studies have shown that regional variations significantly impact blowfly population dynamics, highlighting the importance of region-specific data in medico-legal entomology [15,23]. Detailed studies on the ecological and biological aspects of blowfly activity can provide supplementary evidence in forensic cases, offering insights into the circumstances surrounding the discovery of remains [24]. This is particularly relevant in regions like South Korea, where distinct seasonal and geographical factors can influence insect activity. While global databases like the Global Biodiversity Information Facility (GBIF) and the Korean Natural History Research Information System (NARIS) provide valuable biodiversity data, they often lack the regional and seasonal specificity needed for forensic applications [14,18]. Therefore, there is a pressing need for comprehensive, quantitative data on blowfly species diversity and distribution across well-defined regions and time frames to effectively support forensic investigations.

Globally, forensic research into the distribution and diversity of blowfly species is currently being conducted, with significant contributions from various countries, including the United States [15,19,23], Canada [25,26], Colombia [27], Argentina [28], Switzerland [29,30], Germany [17,31], Spain [32], and Japan [33]. Despite these extensive efforts, research focusing on the forensically significant blowfly species in South Korea remains sparse, with a notable lack of comprehensive regional coverage. Thus, detailed investigations into the blowfly populations across various South Korean regions are required. In this study, we aim to explore the spatio-temporal distribution of forensically relevant blowflies in Gyeongsangnam-do, South Korea. Given the known impact of environmental factors such as temperature, habitat, and geographical location on blowfly species distribution and diversity, this research posits the hypothesis that community composition, species abundance, and diversity will differ across different regions, seasons, and habitats.

## 2. Materials and Methods

### 2.1. Site Description

**Figure 1:**
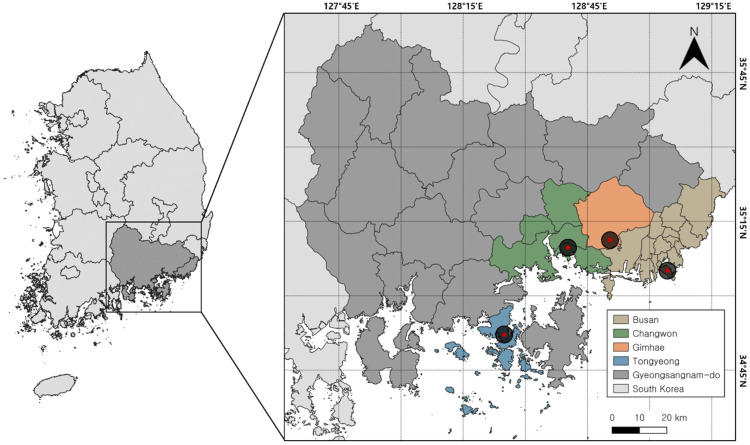
Location of sampling sites by region in Gyeongsangnam-do, South Korea. Black circles represent zones containing all sampling sites for urban and forest habitats within each region.

This study encompasses a comprehensive survey conducted over the span of one year within four distinct regions of Gyeongsangnam-do, South Korea: Busan, Changwon, Gimhae, and Tongyeong (Figure 1). Each region was selected based on its unique geographical characteristics, as delineated below:

Busan (BS): This region is characterized by its mountainous terrain, is bordered by the sea, and includes islands along the eastern and southern coasts, conferring a diverse ecosystem.

Changwon (CW): Distinguished by a mountainous valley landscape, Changwon offers a unique combination of natural topographical features.

Gimhae (GH): This region is notable for its proximity to the South Sea and several rivers, providing a distinctive aquatic and terrestrial interface.

Tongyeong (TY): Tongyeong is a peninsula known for its ria coast, and its landscape is marked by intricate coastlines and varied maritime habitats.

To investigate the difference in species composition and abundance of blowflies based on habitat types within the study regions, the trap deployment sites were classified into two types: urban and forest. Urban habitats were defined by densely populated areas featuring man-made structures, such as roadsides or residential areas. Conversely, the forest habitats were characterized by sparsely populated areas, typically located at the base of mountains or within wooded terrains, devoid of man-made structures. The precise location of each trap was documented using GPS coordinates to ensure accuracy in data collection. The spatial distribution of the selected regions ranged from a minimum distance of approximately 15.3 km to a maximum distance of approximately 64.4 km, highlighting the study’s geographical breadth.

### 2.2. Sampling Procedure

Bait traps, recognized for their efficacy in capturing the broad characteristics of seasonal and regional variations in blowfly populations, were employed as the primary collection method [34]. The survey was conducted twice a month in each region from May 2022 to May 2023. For each sampling event, a total of 10 bait traps were deployed: five in the urban habitats and five in the forest habitats. These traps were placed for a duration of 48 h, with an inter-trap distance of approximately 20–50 m, to ensure spatial independence [35]. New traps were used for each survey to prevent any residual attractant effects. Thus, a total of 960 traps were deployed over the study period.

**Figure 2:**
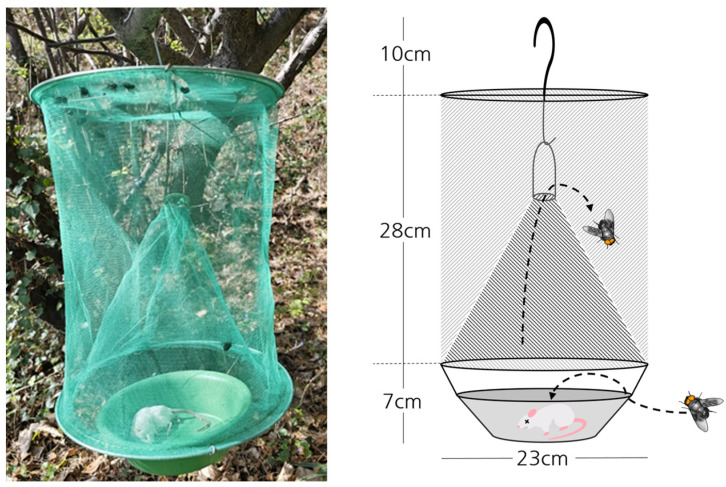
Conical bait trap used in the experiment.

The design of the bait trap consisted of a conical structure equipped with a hook at the apex, suspended 1.5–2 m above ground level (Figure 2). This elevation strategy was adopted to restrict access to ants and other non-target ground insects [34]. As an attractant, thawed fresh small mouse carcasses weighing between 20 and 30 g were employed. These carcasses were brought in vacuum packed at room temperature before being used in the traps.

Given the observed decrease in fly activity during periods of rainfall [11,17,24], efforts were made to schedule trap deployment during dry periods to avoid dilution of the attractant’s scent by rainwater, which may impede blowfly attraction. Upon collection, the blowflies were euthanized using a pyrethroid insecticide and preserved in 70% ethanol for subsequent analysis. Morphological identification of all adult specimens at the species level was conducted using the taxonomic keys provided by Ji [36] and Kanō and Shinonaga [37]. The specimens are preserved in the entomological collection at the Department of Biomedical Sciences, Kosin University, for future reference and study.

### 2.3. Data Analysis

**Figure 3:**
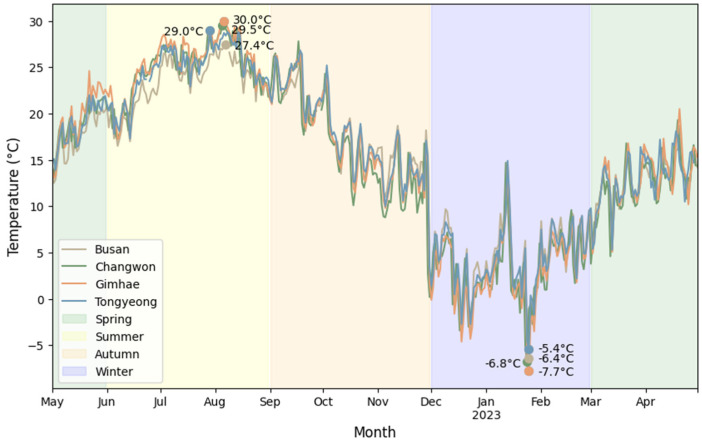
Average daily temperature in the region where traps were installed during the survey period from 2022 to 2023. Plots show the maximum and minimum temperatures for each region.

Blowfly community analysis was segmented by region, season, and habitat, with seasonal divisions according to the climatic patterns of Gyeongsangnam-do, South Korea, as delineated by the official weather website for spring (March to May), summer (June to August), autumn (September to November), and winter (December to February) (Figure 3) [38]. To elucidate the diversity of blowfly species in each season and region, metrics relative abundance, species diversity determined via the Shannon–Wiener index (H’), and species richness calculated through the Margalef index (R) were computed.

To reveal differences in temperature across regions and seasons, a one-way analysis of variance (ANOVA) was performed, followed by a Tukey’s HSD test as a post-hoc test. The average daily temperature was obtained from the Korea Meteorological Administration and was calculated from weather stations up to 10 km from the survey site.

One-way ANOVA was employed to identify regional and seasonal disparities in species diversity and richness. Statistically significant differences were analyzed using post-hoc analysis with Tukey’s HSD test. Furthermore, the Kruskal–Wallis test was utilized to evaluate differences in relative abundance across seasons for the nine species that constituted more than 1% of the total community captured using the traps. Subsequent to significant Kruskal–Wallis test results, the Bonferroni correction was used to adjust the *p*-values obtained from Dunn’s test. Winter was excluded from the statistical analysis due to the zero variance of the data.

The temporal dynamics of species abundance were visualized through a heatmap generated via two-way hierarchical clustering analysis, employing the unweighted pair group method with arithmetic mean (UPGMA) algorithm and Bray–Curtis similarity. Each month throughout the survey period was plotted along the X-axis, while the nine predominant blowfly species were presented along the Y-axis.

Species diversity in different habitats was assessed using the Shannon–Wiener index (H’) and Margalef index (R), employing the same procedures as previously mentioned. Habitat preferences of the dominant species, representing more than 1% of the total community, were examined using a chi-square test of the trap data acquired throughout the study. To provide a more comprehensive view of the distribution patterns of blowflies, the relative abundances of environmental parameters (region and season, habitat) were analyzed using principal component analysis (PCA).

For all statistical tests, the Shapiro–Wilk test was applied to assess data normality, and a *p*-value threshold of <0.05 was considered significant. Data analysis was performed in SPSS (version 23.0) [39] and Python (version 3.12.0) along with the pandas (version 2.1.3), numpy (version 1.26.1), scipy (version 1.11.3), matplotlib (version 3.8.1), and seaborn (version 0.13.0) libraries.

## 3. Results

### 3.1. Seasonal and Regional Variations in Blowfly Abundances and Diversity

Throughout the investigation period, a total of 3470 adult blowflies, encompassing 13 species across five genera, were collected (Table 1). Notably, no specimens were gathered during the winter season. *Lucilia porphyrina* emerged as the most prevalent species, with 1291 individuals accounting for 37.2% of the total collection, followed by *Chrysomya pinguis* (956 individuals representing 27.6%), *Lucilia sericata* (263 individuals, 7.6%), and *Lucilia illustris* (247 individuals, 7.1%).

#### 3.1.1. Analysis of Temperature Differences

**Figure 4:**
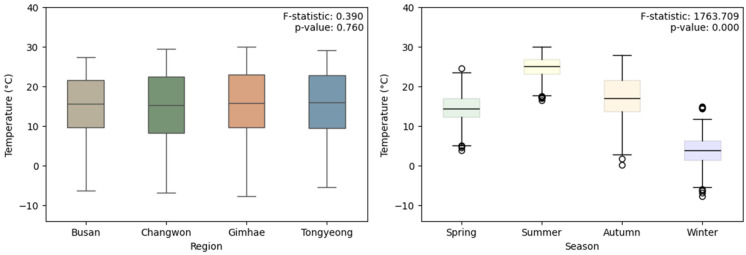
Box plots showing differences in average temperature by region and season (*p*-value for regional differences: 0.760, *p*-value for seasonal differences: <0.001).

The analysis of temperature differences between regions and seasons showed that there was no significant difference in the mean temperature between regions (F = 0.390, df = 3, *p* = 0.760), but there was a significant difference in the mean temperature between seasons (F = 1763.709, df = 3, *p* < 0.001). Furthermore, post-hoc analysis revealed that the mean temperature significantly differed between all pairs of seasons (*p* < 0.001) (Figure 4).

#### 3.1.2. Analysis of Blowfly Species Relative Abundance

**Figure 5:**
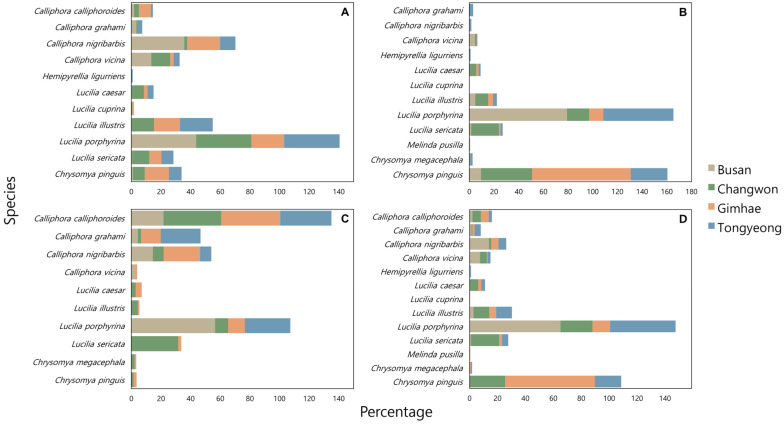
Relative abundance of blowflies in each region during each season. (**A**): Spring, (**B**): Summer, (**C**): Autumn, (**D**): All seasons.

Analysis of the relative abundance of blowfly species across different regions and seasons revealed significant seasonal variations in species distribution (Figure 5). In the spring, *Calliphora nigribarbis* and *L. porphyrina* exhibited the highest abundance in Gimhae, constituting 22.1% of all blowflies, whereas only *L. porphyrina* was predominantly abundant in Busan, Changwon, and Tongyeong, with proportions of 43.7%, 37.4%, and 37.4%, respectively. During the summer months, *Ch. pinguis* exhibited the highest abundance in Gimhae and Changwon at 80.0% and 41.5%, respectively, while *L. porphyrina* was most prevalent in Busan and Tongyeong, accounting for 79.1% and 56.8%, respectively. In autumn, *Calliphora calliphoroides* was notably abundant in Gimhae, Changwon, and Tongyeong at 39.8%, 39.0%, and 34.6%, respectively, with *L. porphyrina* maintaining the highest abundance in Busan at 56.5%.

#### 3.1.3. Analysis of Species Diversity

**Figure 6:**
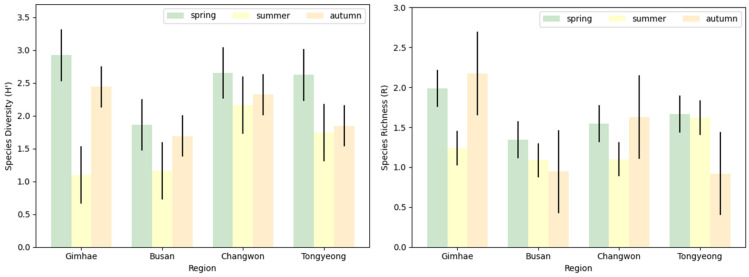
Variations in the species diversity (H’) and richness (R) of blowflies between different regions and seasons. Vertical bars represent standard deviation.

Although statistical analysis of species diversity indicated no significant differences between regions (F = 1.142, df = 3, *p* = 0.389), a notable variation between seasons (F = 4.817, df = 2, *p* = 0.038) was observed (Figure 6). Species diversity peaked in spring and was lowest in summer. Species diversity in autumn did not significantly differ from that in spring (*p* = 0.398) or summer (*p* = 0.246); however, there was a significant disparity in species diversity between spring and summer (*p* = 0.031). Species richness showed no significant variation among regions (F = 1.749, df = 3, *p* = 0.234) or seasons (F = 0.873, df = 2, *p* = 0.450).

### 3.2. Seasonal Differences in Blowfly Abundance

The Kruskal–Wallis test was applied to examine the seasonal relative abundances of the nine predominant captured blowfly species that constituted more than 1% of the total community (Table 2). The results indicated significant seasonal variations in the abundances of four species. The abundance of *C. calliphoroides*, *Calliphora grahami*, and *Ch. pinguis* exhibited significant seasonal differences between summer and autumn (*p* = 0.004, *p* = 0.043 and *p* = 0.013, respectively). The abundances of *C. nigribarbis* displayed notable seasonal differences between spring and summer (*p* = 0.042).

**Figure 7:**
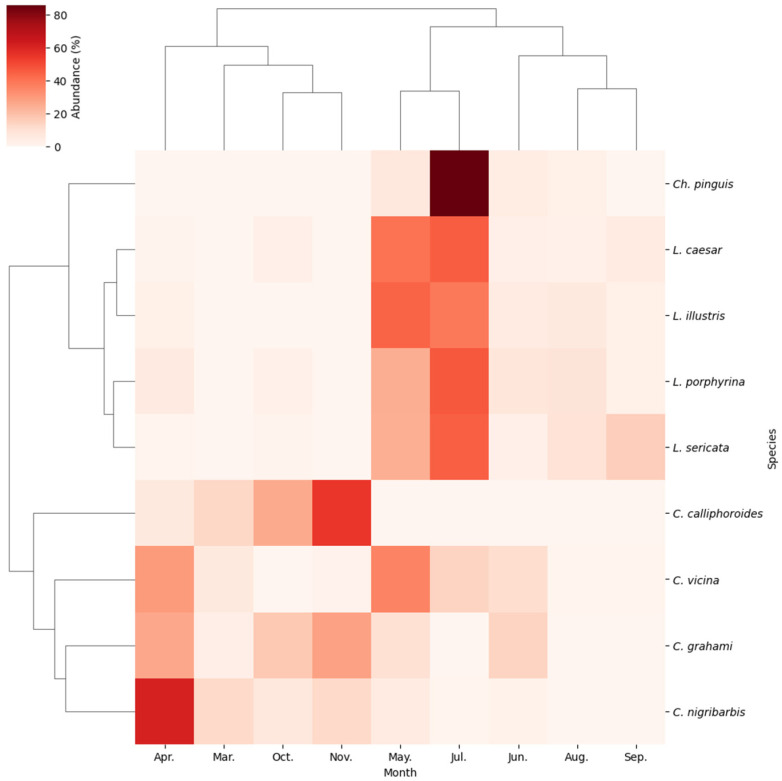
Two-way hierarchical clustering analysis based on the UPGMA algorithm and Bray–Curtis similarity, and a heatmap representing monthly species abundance. The dendrogram on the left shows a cluster of the nine dominant species. The upper dendrogram shows a cluster of the highest species abundances collected each month. Darker shading in the cells of the array indicates higher abundances.

Two-way hierarchical clustering and heatmap analyses of the community composition of blowflies revealed distinct monthly correlations among the nine species (Figure 7). At the genus level, the clustering resulted in a discernible separation into three groups: one comprising four *Calliphora* species, one *Chrysomya*, and four *Lucilia*. Monthly clustering identified that March, April, October, and November, typically the early spring and late autumn months, formed distinct subgroups, while species in May through September clustered into another subgroup, indicating seasonal influences on community composition.

### 3.3. Habitat Preferences and Environmental Influences on Blowfly Species Distribution

The investigation into species diversity and richness across different habitats revealed that, in urban habitats, species diversity was 2.895 (H′), but was 2.019 (H′) in forest habitats, indicating a higher diversity in urban environments. Conversely, species richness presented similar values between habitats (1.153 (R) in urban habitats and 1.027 (R) in forest settings). These results suggest a distinction in habitat suitability for various blowfly species.

Further analysis identified specific habitat preferences among the blowfly species (Table 3). Five species demonstrated a preference towards urban habitats, with *C. calliphoroides* and *L. sericata* showing a significantly higher preference than that of other species (*p* < 0.001). Additionally, *Calliphora vicina* favored urban settings, albeit without statistical significance. Conversely, three species exhibited a preference for forest habitats, with *L. porphyrina* and *Ch. pinguis* displaying a marked inclination towards these environments (*p* < 0.001). *Calliphora nigribarbis* did not exhibit a clear preference for either habitat type.

**Figure 8:**
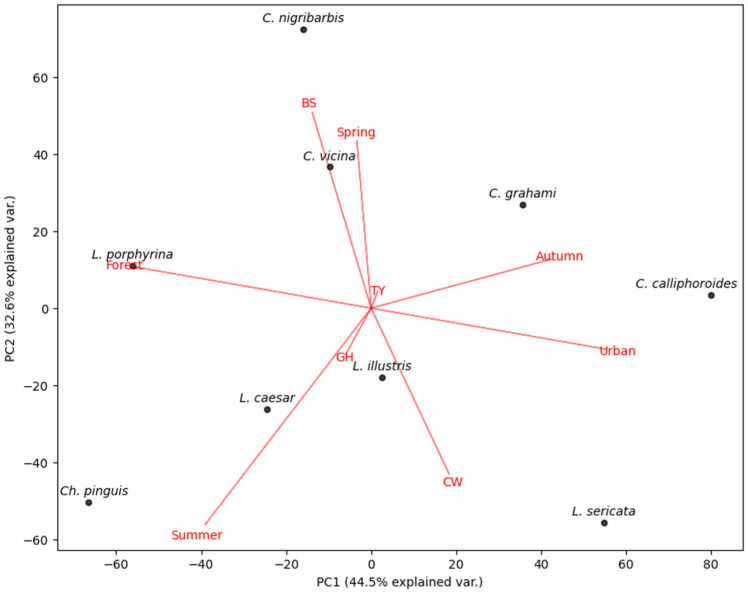
PCA of relative abundance of environmental parameters (region, season, habitat). BS: Busan, CW: Changwon, GH: Gimhae, TY: Tongyeong.

The distribution of blowflies is clearly reflected in the PCA, which is related to environmental influences such as season and habitat (Figure 8). The PCA (PC1 and PC2 accounting for 77.1% of the variation) showed that spring and autumn contrasted clearly with summer. Species belonging to the genus *Calliphora* were found to be associated with spring and autumn, while *Ch. pinguis*, *Lucilia caesar*, and *L. illustris* were generally associated with summer. The vectors for Gimhae and Tongyeong were found to have no significant explanatory power compared to the other vectors.

## 4. Discussion

This research presents the first comprehensive analysis of blowfly communities within the Gyeongsangnam-do region. This study successfully identified 14 distinct blowfly species, with *L. porphyrina* (37.2%), *Ch. pinguis* (27.6%), *L. sericata* (7.6%), and *L. illustris* (7.1%) emerging as the predominant species. Notably, the composition of the blowfly community did not exhibit significant variations between different sites within the region. However, seasonal distinctions were apparent, with community composition differing between every season apart from winter. This seasonal variation in community composition underscores the influence of climatic factors on blowfly activity and diversity. Upon analyzing climatic factors, we found no significant differences in the annual average daily temperatures among the four study regions. This suggests that the cause affecting the distribution of blowflies in the region is not the difference in daily average temperature. However, significant differences in average temperature between seasons were confirmed. This supports the findings of this study that the seasonal division of the four regions was appropriately differentiated according to daily average temperature and that the distribution of blowflies is more influenced by seasonal factors governing different environmental conditions.

We analyzed the distribution of blowflies by considering both species diversity and species richness to better understand their response to ecological and environmental changes in the study area. In all regions, species diversity decreased during the summer, which may be due to the dominance of *L. porphyrina* and *Ch. pinguis*, which accounted for a significant proportion of the total individuals collected during this period. Such a high prevalence of these two species in the summer suggests they have a possible competitive advantage or preference for the environmental conditions present during this season.

### 4.1. Ecological Niches and Seasonal Distribution of Forensic Blowflies

Our findings reveal a distinct season-dependent prevalence of the genus *Calliphora*, which was more abundant in spring and autumn than in summer. Specifically, *C. calliphoroides* (also known as *Triceratopyga calliphoroides*) was absent during summer and winter, and its highest relative abundance occurred in autumn. This pattern underscores its pronounced seasonal preference. The genus *Calliphora* is typically found colonizing remains in significant numbers during the cool seasons, such as autumn, winter, and spring, with the exact timing varying by geographic location [16,19,26,40]. In Korea, *Calliphora* species have been frequently observed on carcasses in spring and autumn [7]. Forensic autopsy data obtained in Korea corroborated our findings, with species such as *C. calliphoroides*, *C. grahami*, *C. nigribarbis*, and *C. vicina* predominantly identified in these seasons [41], highlighting their forensic significance in determining the mPMI.

The genus *Lucilia* was present in spring, summer, and autumn, with the most abundant during the summer. *Lucilia porphyrina*, the dominant species throughout Gyeongsangnam-do, exhibited limited abundance in metropolitan autopsies [41]. Notably, *L. porphyrina* thrives in Southeast Asia and tropical and subtropical mountainous regions with higher altitudes [42], suggesting that geographical and latitudinal differences may influence its distribution. Other *Lucilia* species mirrored the seasonal pattern of *L. porphyrina*, with *L. sericata* being particularly prevalent in domestic autopsies, underscoring its forensic importance in these cases [41].

Within the genus *Chrysomya*, both *Ch. pinguis* and *Ch. megacephala* were collected, but their abundances varied significantly. *Chrysomya pinguis* emerged as the second most abundant species, exhibiting a strong association with summer. Although *Ch. megacephala* is globally recognized as forensically significant [43], it constituted only 0.4% of the total population in this study. *Chrysomya pinguis* has a notable presence across various Asian countries [44,45,46] and is a key forensic species in Korea that is frequently found on carcasses and in autopsies across spring, summer, and autumn [6,7,41,47], which is further supported by its high abundance in this study. The differential abundances of *Ch. pinguis* and *Ch. megacephala* may result from ecological niche differentiation, with potential interspecific competition for limited resources [37,46,48]. This competition might lead to *Ch. megacephala* being ecologically displaced by *Ch. pinguis*. Alternatively, Yang and Shiao [46] proposed that temperature differentiation might influence the ecological niches of these species, but our study did not obtain constant abundance data to fully elucidate seasonal distribution differences only for those species.

### 4.2. Ecological Niches and Habitat Preferences of Forensic Blowflies

In this study, species within the *Calliphora* genus exhibited a general preference for urban habitats. *Calliphora calliphoroides* and *C. grahami* demonstrated a clear bias toward urban environments, while *C. vicina* was more frequently found in urban habitats, albeit without statistical significance. Despite *C. vicina*’s known association with urban habitats, regional and environmental variations may influence its lifecycle dynamics [49,50,51]. Altitude has been documented to affect blowflies’ seasonal distribution and oviposition behavior [33,52]. Although no significant habitat preference was noted for *C. nigribarbis* in this study, previous research indicates its tendency to migrate to higher altitudes during summer for breeding, returning to lower altitudes in autumn [33,53]. In addition, Kimura [54] discussed the migration patterns of *C. nigribarbis* and *C. grahami* in the context of univoltine life cycles, distinguishing between upward and downward migration, emphasizing the possibility of diapause-associated migration of low-altitude individuals. In particular, only some of the high-altitude individuals were moving at low altitudes; therefore, whether this migration can be classified as dispersion remains controversial. The similarities between migration and dispersion complicate identifying a clear definition, underscoring the need for further research on these species’ elevation-based life cycles and migration behaviors.

The *Lucilia* genus displayed varied habitat preferences among species. *Lucilia caesar* and *L. porphyrina* showed a predilection for forested habitats, while *L. sericata* and *L. illustris* favored urban settings. *Lucilia porphyrina*’s migration from the highlands to the lowlands for hibernation, followed by returning to the highlands for oviposition in summer [42], may explain its forest-biased habitat preference, as its lifecycle is predominantly spent in woodlands during summer. Here, *L. sericata* exhibited a strong preference for urban habitats and is known as an urban species with human affinities; it has been found in many autopsies in domestic indoor spaces [41,49,55,56]. *Lucilia caesar* and *L. illustris* are known to be morphologically similar sister species [57,58]; the habitat distribution in this study showed conflicting results. *Lucilia caesar* is generally known to be abundant in shady and sparsely populated forested habitats, where its ecology is influenced by low temperature and high humidity factors [59,60,61]. These habitat differences may be related to niche partitioning. As carcasses are a limited and transient resource, carrion ecosystems are characterized by intense resource competition [61,62]. Therefore, it is possible that the two species have diverged to adapt to different habitats to minimize direct competition.

*Chrysomya pinguis* exhibited a preference for forested habitats, aligning with observations of its prevalence on corpses in less densely populated regions [56] and its abundance in mountainous areas during summer [53]. This species likely dominates high-land or forest habitats in summer, with potential spring and autumn migrations to low-land areas, possibly related to the diapause-associated migration behaviors described for *C. nigribarbis* and *C. grahami* [54,63].

### 4.3. Ecological Insights and Forensic Dynamics of Blowflies

This year-long investigation in Gyeongsangnam-do, South Korea, yielded fundamental forensic entomological data, laying the groundwork for enhancing forensic case studies in this region. The species cataloged in this research have been observed in both domestic animal carcasses and human autopsies, affirming their forensic relevance within South Korea [6,7,41]. The local blowfly data obtained by this study underscores the necessity for a comprehensive nationwide blowfly distribution dataset for refining forensic insect estimation techniques in future investigations.

This study was limited to blowfly colonization during the early stages of carcass decomposition because the bait used to replace the odor of the decomposing carcass was only allowed to colonize for two days after the onset of decomposition. Despite this limitation, our findings support the hypothesis that different blowfly species exhibit distinct habitat preferences, as well as specific seasonal and geographical distribution patterns. These observed patterns are instrumental for forensic entomologists to determine the geographical origin of insects found on a corpse, assess potential corpse movement, and deduce the location of the crime. Understanding the ecological niches and behavior of blowfly species can significantly enhance the utility of forensic entomology in criminal investigations. By recognizing species-specific habitat preferences and their seasonal and geographical distribution, forensic entomologists can draw inferences about the environmental conditions surrounding a crime scene [50]. This information can help identify the likely locations where a body may have been moved from or to, providing critical evidence in crime scene reconstruction [18].

It is important to recognize that using mouse cadavers cannot perfectly replicate the condition of human remains. The differences between small mammals, which are typically hairy, and the larger human body, which has less body hair and is often clothed, complicate direct comparisons of research results to actual forensic cases. Nevertheless, insects collected from experiments using animal carcasses have also been found during human autopsies [6,7,8,9,22,41]. Utilizing animal carcasses for research allows for stringent control of experimental conditions and the repetition of experiments with fewer ethical and legal constraints. Therefore, studies involving animal carcasses can sufficiently support the findings obtained from human cadaver studies.

The data from this study also highlight the importance of localized research in building a robust forensic framework. The specific blowfly species identified, as well as their patterns of colonization, offer valuable insights into regional biodiversity and ecological dynamics. Detailed regional studies are crucial for developing accurate forensic models that can be applied to various scenarios, ultimately improving the precision of forensic analyses. Beyond immediate forensic applications, the ecological information gained from this study contributes to a broader understanding of blowfly ecology and their role in the decomposition process. The observed habitat preferences and distribution patterns can inform future studies on blowfly behavior and their interactions with the environment, which is vital for both forensic science and ecological research.

## 5. Conclusions

This investigation provides essential data on the spatio-temporal distribution of blowfly species in Gyeongsangnam-do, South Korea, emphasizing the need for comprehensive regional datasets. The findings highlight the significance of species diversity, habitat preferences, and ecological niches in forensic entomology, offering valuable insights for crime scene investigations and advancing the field’s scientific foundation. Future research should focus on expanding the dataset to include various stages of decomposition and different environmental conditions to further refine forensic entomological methods and applications.

## Figures and Tables

**Table 1:** Total Number and Percentage of Collected Blowfly Species During the Investigation Period.

Species	Region				Habitat		Total Collected	Percent (%)
	BS	CW	GH	TY	Urban	Forest		
*Calliphora calliphoroides* (Rohdendorf, 1931)	22	64	46	12	115	29	144	4.1
*Calliphora grahami* (Aldrich, 1930)	16	6	13	22	37	20	57	1.6
*Calliphora nigribarbis* (Vollenhoven, 1863)	151	17	43	28	73	166	239	6.9
*Calliphora vicina* (Robineau-Desvoidy, 1830)	82	52	4	10	66	82	148	4.3
*Hemipyrellia ligurriens* (Wiedemann, 1830)	-	2	2	3	2	5	7	0.2
*Lucilia caesar* (Linnaeus, 1758)	3	63	18	13	25	72	97	2.8
*Lucilia cuprina* (Wiedemann, 1830)	1	2	1	-	2	2	4	0.1
*Lucilia illustris* (Meigen, 1826)	31	118	41	57	140	107	247	7.1
*Lucilia porphyrina* (Walker, 1856)	707	242	104	238	207	1084	1291	37.2
*Lucilia sericata* (Meigen, 1826)	11	213	16	23	245	18	263	7.6
*Melinda pusilla* (Villeneuve, 1927)	-	-	4	-	4	-	4	0.1
*Chrysomya megacephala* (Fabricius, 1794)	-	4	4	5	7	6	13	0.4
*Chrysomya pinguis* (Walker, 1858)	63	269	528	96	188	768	956	27.6
Total	1087	1052	824	507	1111	2359	3470	100.0

**Table 2:** Seasonal differences in the relative abundances of blowfly species using Kruskal–Wallis and Dunn’s tests.

Species	Kruskal–Wallis Test		Dunn’s Test		
	Season		Spring vs. Summer	Spring vs. Autumn	Summer vs. Autumn
	F	*p*			
*C. calliphoroides*	10.203	0.006 *	NS	NS	0.004
*C. grahami*	6.048	0.049 *	NS	NS	0.043
*C. nigribarbis*	7.449	0.024 *	0.042	NS	NS
*C. vicina*	5.365	0.068 ^NS^	NA	NA	NA
*L. caesar*	1.389	0.499 ^NS^	NA	NA	NA
*L. illustris*	4.786	0.091 ^NS^	NA	NA	NA
*L. porphyrina*	1.423	0.491 ^NS^	NA	NA	NA
*L. sericata*	0.618	0.734 ^NS^	NA	NA	NA
*Ch. pinguis*	8.144	0.017 *	NS	NS	0.013

*, significant difference; NS, non-significant difference; NA, not applied; Dunn’s test, which was applied for only two seasons, and Bonferroni correction (0.05 > *p*-value * 3 tests) was required.

**Table 3:** Habitat preferences of blowflies collected in Gyeongsangnam-do.

Species	Urban:Forest	Chi-Squared
*C. calliphoroides*	1:0.38	χ^2^ = 23.937, df = 1, *p* < 0.001 *
*C. grahami*	1:0.48	χ^2^ = 8.471, df = 1, *p* = 0.004 *
*C. nigribarbis*	1:1	NA
*C. vicina*	1:0.81	χ^2^ = 1.774, df = 1, *p* = 0.183 ^NS^
*L. caesar*	0.62:1	χ^2^ = 4.016, df = 1, *p* = 0.045 *
*L. illustris*	1:0.75	χ^2^ = 4.719, df = 1, *p* = 0.030 *
*L. porphyrina*	0.54:1	χ^2^ = 50.562, df = 1, *p* < 0.001 *
*L. sericata*	1:0.25	χ^2^ = 47.459, df = 1, *p* < 0.001 *
*Ch. pinguis*	0.57:1	χ^2^ = 18.847, df = 1, *p* < 0.001*

*, significant difference; NS, non-significant difference; NA, not applied.

## Data Availability

The original contributions presented in the study are included in the article; further inquiries can be directed to the corresponding authors.
